# Therapeutic Indications of Pembrolizumab in Eight Common Cancers: Current Evidence and Future Directions

**DOI:** 10.1002/cnr2.70234

**Published:** 2025-07-10

**Authors:** Raghed Mansour, Alghaidaq Shreba, Karam Khaddour, Michael Georgeos, Zuheir Alshehabi

**Affiliations:** ^1^ Faculty of Medicine, Tishreen University Lattakia Syrian Arab Republic; ^2^ Cancer Research Center, Tishreen University Lattakia Syrian Arab Republic; ^3^ Dana‐Farber Cancer Institute, Harvard Medical School Boston Massachusetts USA; ^4^ Department of Oncology Tishreen University Hospital Lattakia Syrian Arab Republic; ^5^ Department of Pathology Tishreen University Hospital Lattakia Syrian Arab Republic

**Keywords:** cancer treatment, immune checkpoint inhibitor, immunotherapy, pembrolizumab, therapeutic indications

## Abstract

**Background:**

Pembrolizumab is a monoclonal antibody that inhibits the programmed death‐1 (PD‐1) receptor pathway, which has increasingly been implicated in cancer treatment regimens. Since its first approval for melanoma in 2014, many trials have investigated the efficacy and safety of this new drug in different cancers. In this review, we discuss the therapeutic advances achieved with pembrolizumab in the management of eight cancers that are associated with a relatively poor prognosis. We also report the FDA approvals of this drug, highlighting promising ongoing trials and potential aspects for future research.

**Recent Findings:**

Numerous trials have demonstrated robust anti‐cancer effects, high response rates, and a favorable safety profile of pembrolizumab monotherapy or its combination in different lines and treatment settings. With the encouraging survival benefits of this treatment in advanced/metastatic disease, there has been an increasing tendency to explore its therapeutic potential in early‐stage disease. Thus, pembrolizumab was effectively added to the standard neoadjuvant chemotherapy regimen for resectable TNBC and NSCLC, followed by adjuvant pembrolizumab monotherapy after resection. Similar positive results were found with the adjuvant administration of pembrolizumab after surgery in resectable RCC and melanoma. Pembrolizumab has also been recently studied in locally advanced resectable gastric and gastroesophageal junction adenocarcinoma as well as early‐stage estrogen receptor‐positive/human epidermal growth factor receptor 2‐negative breast cancer as a neoadjuvant‐adjuvant regimen.

**Conclusions:**

The advent of immunotherapeutic agents such as pembrolizumab has unprecedently altered cancer therapy regimens; however, future research efforts should address the need for biomarkers that could better identify patients who would most likely respond to such therapy and investigate new combinations that could overcome resistance to immunotherapy.

## 1 Introduction

Pembrolizumab, a humanized monoclonal antibody that blocks programmed death receptor‐1 (PD‐1), was first introduced in 2014 as a new option for melanoma patients with metastatic disease that had progressed on prior therapy [1]. Soon after, Le et al. [2] established the benefit of mismatch‐repair status in predicting efficacy outcomes for immune checkpoint blockade with pembrolizumab, as somatic mutations that frequently occur in microsatellite instability‐high (MSI‐H) cancers were hypothesized to generate immunogenic antigens that potentiate immune response. Evidence supporting this finding accumulated for both colorectal [3] and non‐colorectal [4] cancers and eventually outlined the approval of pembrolizumab as the first tumor agnostic treatment for microsatellite instability‐high/mismatch‐repair‐deficient cancers (MSI‐H/dMMR) of any type in both adults and pediatric populations after progression on prior therapy [5]. In the past decade, pembrolizumab has been increasingly investigated in various cancers for which no sufficiently effective treatments were available (Table 1).

**TABLE 1:** A table of clinical trials investigating pembrolizumab in TNBC, NSCLC, melanoma, RCC, UC, HNSCC, EC, CRC and their respective FDA approvals.

Cancer type	Trial	Patients included	# of patients	Intervention	Results OS, PFS, DFS, EFS (months)	FDA approvals
TNBC	KEYNOTE‐522 [6]	Previously untreated stage II or stage III TNBC	1174	Neoadjuvant pembrolizumab + chemotherapy vs. placebo + neoadjuvant chemotherapy^a^	Percentage of patients with a PCR: (64.8% vs. 51.2%)	Approved for high‐risk, early‐stage, TNBC in combination with chemotherapy as neoadjuvant treatment, and then continued as a single agent as adjuvant treatment after surgery [7]
	KEYNOTE‐355 [8]	Locally recurrent inoperable or metastatic TNBC	847	Pembrolizumab + chemotherapy vs. placebo + chemotherapy	Median OS in patients with CPS of 10 or more: (23.0 vs. 16.1)	In combination with chemotherapy for the treatment of patients with locally recurrent unresectable or metastatic TNBC whose tumors express PD‐L1 (CPS ≥ 10) as determined by an FDA approved test [9]
NSCLC	KEYNOTE‐024 [10]	Previously untreated advanced NSCLC with TPS ≥ 50% and no EGFR or ALK mutations	305	Pembrolizumab vs. platinum‐based chemotherapy	Median PFS: (10.3 vs. 6.0) OS rate at 6 months: (80.2% vs. 72.4)	First line treatment for patients with metastatic NSCLC whose tumors have high PD‐L1 expression (TPS ≥ 50%) as determined by an FDA‐approved test, with no EGFR or ALK genomic tumor aberrations, and no prior systemic chemotherapy treatment for metastatic NSCLC [11]

KEYNOTE‐042 [12]	Previously untreated locally advanced or metastatic NSCLC with TPS ≥ 1% and no EGFR or *ALK* mutations	1274	Pembrolizumab vs. platinum‐based chemotherapy	Median OS: (20.0 vs. 12.2, 17.7 vs. 13.0, and 16.7 vs. 12.1) in TPS populations (≥ 50%, ≥ 20%, and ≥ 1%), respectively	First‐line treatment of patients with stage III NSCLC who are not candidates for surgical resection or definitive chemoradiation or metastatic NSCLC. Patients' tumors must have no *EGFR* or *ALK* genomic aberrations and express PD‐L1 (Tumor Proportion Score [TPS] ≥ 1%) determined by an FDA‐approved test [13]

KEYNOTE‐189 [14]	Untreated metastatic non‐squamous NSCLC without *EGFR* or *ALK* mutations	616	Pembrolizumab + chemotherapy vs. placebo + chemotherapy	OS at 12 months: (69.2% vs. 49.4%), OS benefit was observed in all PD‐L1 TPS subgroups Median PFS: (8.8 vs. 4.9)	In combination with pemetrexed and platinum as first‐line treatment of patients with metastatic, non‐squamous NSCLC, with no *EGFR* or *ALK* genomic tumor aberrations [15]

KEYNOTE‐407 [16]	Untreated metastatic, squamous NSCLC	559	Pembrolizumab + chemotherapy vs. placebo + chemotherapy	Median OS: (15.9 vs. 11.3), OS benefit was consistent regardless of PD‐L1 expression Median PFS: (6.4 vs. 4.8)	In combination with carboplatin and either paclitaxel or nab‐paclitaxel as first‐line treatment of metastatic squamous NSCLC [17]

KEYNOTE‐010 [18]	Previously treated advanced NSCLC with TPS ≥ 1%	1034	Pembrolizumab 2 mg/kg or 10 mg/kg vs. docetaxel	Median OS: 10.4 for pembrolizumab 2 mg/kg, 12.7 for pembrolizumab 10 mg/kg, and 8, 5 for docetaxel	For patients with metastatic NSCLC whose tumors express PD‐L1 (TPS ≥ 1%), with disease progression on or after platinum‐containing chemotherapy. Patients with *EGFR* or *ALK* genomic tumor aberrations should have disease progression on FDA‐approved therapy for these aberrations prior to receiving pembrolizumab [19]

PEARLS/KEYNOTE‐091 [20]	Completely resected, stage IB, II, or IIIA NSCLC	1177	Adjuvant Pembrolizumab vs. placebo Both arms were given adjuvant chemotherapy when recommended	Median disease‐free survival in the overall population: (53.6 vs. 42.0)	Adjuvant treatment following resection and platinum‐based chemotherapy for stage IB (T2a ≥ 4 cm), II, or IIIA NSCLC [21]

KEYNOTE‐671 [22]	Resectable, stage II or III NSCLC	797	Neoadjuvant pembrolizumab or placebo plus chemotherapy followed by surgery and adjuvant pembrolizumab or placebo	24‐month event‐free survival: 62.4% in pembrolizumab group vs. 40.6% in placebo group MPR: 30.2% vs. 11.0% CPR: 18.1% vs. 4.0%	With platinum‐containing chemotherapy as neoadjuvant treatment, and with continuation of single‐agent pembrolizumab as post‐surgical adjuvant treatment for resectable (tumors ≥ 4 cm or node positive) NSCLC [23]
Melanoma	KEYNOTE‐054 [24]	Resected, high‐risk, stage III melanoma	1019	Adjuvant pembrolizumab vs. placebo	12‐month RFS: (75.4% vs. 61.0%) Distant metastasis‐free survival: (65.3% vs. 49.4%) [25]	Adjuvant treatment of patients with melanoma with involvement of lymph node(s) following complete resection [26]
	KEYNOTE‐716 [27]	Resected, high‐risk, stage IIB or IIC melanoma	976	Adjuvant pembrolizumab vs. placebo	15% vs. 24% had a first recurrence or died at second interim analysis	Adjuvant treatment of adult and pediatric (≥ 12 years of age) patients with stage IIB or IIC melanoma following complete resection [28]
	KEYNOTE‐006 [29]	Unresectable stage III or IV melanoma	834	Pembrolizumab (q2wk) vs. pembrolizumab (q3wk) vs. ipilimumab	6‐month PFS rates: (47.3% vs. 46.4% vs. 26.5%) 12‐month OS rates: (74.1% vs. 68.4% vs. 58.2%)	First‐line treatment of patients with unresectable or metastatic melanoma regardless of *BRAF* status [30]
RCC	CLEAR [31]	Advanced RCC, no prior systemic therapy	1069	First‐line lenvatinib with either Pembrolizumab or Everolimus vs. Sunitinib alone	Lenvatinib + Pembrolizumab vs. Sunitinib median PFS: (23.3 vs. 9.2) Median follow‐up OS: (33.7 vs. 33.4) Median OS: (not reached for both)	First‐line treatment in combination with lenvatinib for adult patients with advanced RCC [32]
	KEYNOTE‐426 [33]	Untreated locally advanced/metastatic ccRCC	861	Pembrolizumab+ Axitinib vs. Sunitinib	Median PFS: (15.4 vs. 11.1) Median OS: (not reached vs. 35.7)	First‐line treatment in combination with axitinib for patients with advanced renal cell carcinoma [34]
	KEYNOTE‐564 [35]	Intermediate‐high/high‐risk resected RCC	994	Adjuvant pembrolizumab vs. placebo	DFS after 30 months was better with pembrolizumab (HR 0.63 [95% CI 0.50–0.80])	Adjuvant treatment of patients with renal cell carcinoma at intermediate‐high or high risk of recurrence following nephrectomy, or following nephrectomy and resection of metastatic lesions [36]
UC	KEYNOTE‐045 [37]	Platin refractory metastatic/locally advanced UC	521	Second‐line Pembrolizumab vs. cytotoxic chemotherapy	Median OS: (10.1 vs. 7.2) 48‐month PFS: (9.5% vs. 2.7%)	Second‐line treatment for patients with locally advanced or metastatic urothelial carcinoma who have disease progression during or following platinum‐containing chemotherapy or within 12 months of neoadjuvant or adjuvant treatment with platinum‐containing chemotherapy [38]

KEYNOTE‐052 [37] (Phase II)	Cisplatin‐ineligible patients with metastatic UC	370	First‐line Pembrolizumab	Median OS (11.3) Median PFS (2.5)	Accelerated approval of Pembrolizumab for first‐line treatment of patients with locally advanced or metastatic urothelial carcinoma who are ineligible for cisplatin‐containing chemotherapy [38]

EV‐103 cohort K [39]	Previously untreated cisplatin‐ineligible patients with locally advanced or metastatic UC	149	Enfortumab vedotin + pembrolizumab vs. Enfortumab vedotin alone	Confirmed ORR (64.5% vs. 45.5%) Median DOR (not reached vs. 13.2)	Accelerated approval to first‐line enfortumab vedotin + pembrolizumab for cisplatin‐ineligible patients with locally advanced/metastatic UC [40]

EV‐302 [41]	Cisplatin‐eligible Patients with previously untreated locally advanced/metastatic UC	886	Enfortumab vedotin + pembrolizumab vs. carboplatin‐containing chemotherapy	Median PFS (12.5 vs. 6.3) Median OS (31.5 vs. 16.5)	First‐line Enfortumab vedotin + pembrolizumab for patients with locally advanced or metastatic UC [42]

KEYNOTE‐057 cohort A [43] (phase II)	BCG‐unresponsive high‐risk NMIBC with CIS with or without papillary tumors, ineligible for cystectomy.	96	Pembrolizumab monotherapy	CRR was 41% after 36.4 months at first evaluable assessment	The treatment of patients with BCG‐unresponsive, high‐risk NMIBC with CIS with or without papillary tumors who are ineligible for or have elected not to undergo cystectomy [44]
HNSCC	KEYNOTE‐040 [45]	Recurrent/metastatic HNSCC who have progressed on platinum	495	Pembrolizumab vs. Methotrexate, docetaxel, or cetuximab	Median OS: (8.4 vs. 6.9) % of patients with grade 3 or worse TrAE: (13% vs. 36%)	After progression on or following platinum‐containing therapies, regardless of PD‐L1 expression [46]

KEYNOTE‐048 [47]	Untreated locally incurable recurrent or metastatic HNSCC	882	Pembrolizumab alone or with chemotherapy vs. cetuximab with chemotherapy	Median OS for pembrolizumab alone vs. control in patients with CPS ≥ 20, CPS ≥ 1: (14.9 vs. 10.7), (12.3 vs. 10.3), respectively Median OS for pembrolizumab with chemotherapy vs. cetuximab with chemotherapy in the total population: (13.0 vs. 10.7)	First‐line treatment of patients with metastatic or unresectable recurrent HNSCC, in combination with platinum and fluorouracil (FU) for all patients and as a single agent for patients whose tumors express PD‐L1 (CPS ≥ 1) as determined by an FDA‐approved test [48]
EC	KEYNOTE‐158 [49] (Phase II)	Advanced EC, progressed on previous standard therapy	90	Pembrolizumab monotherapy	ORR: 48% Median DOR: not reached Median PFS: 13.1 Median OS: not reached	As a single agent for patients with advanced endometrial carcinoma that is MSI‐H or dMMR, as determined by an FDA‐approved test, who have disease progression following prior systemic therapy in any setting and who are not candidates for curative surgery or radiation [50]

Study 309‐KEYNOTE‐775 [51]	Advanced, recurrent, or metastatic EC previously treated with platin	827	Lenvatinib plus pembrolizumab vs. chemotherapy	Median PFS, and OS in the pMMR population: (6.6 vs. 3.8), and (17.4 vs. 12.0), respectively	In combination with lenvatinib for patients with advanced EC that is not MSI‐H/dMMR, who have disease progression following prior systemic therapy in any setting and are not candidates for curative surgery or radiation [52]
CRC	KEYNOTE‐177 [53]	Previously untreated locally confirmed MSI‐H/dMMR stage IV CRC	307	Pembrolizumab vs. chemotherapy	Median OS: (not reached vs. 36.7) Median PFS: (16.5 vs. 8.2)	First‐line treatment of patients with unresectable or metastatic MSI‐H/dMMR colorectal cancer [54]

Abbreviations: CIS, carcinoma in situ; CPS, combined positive score; CRC, colorectal cancer; CRR, complete response rate; DFS, disease‐free survival; dMMR, mismatch‐repair‐deficient; DOR, duration of response; EC, endometrial cancer; EFS, event‐free survival; HNSCC, head and neck squamous cell carcinoma; MPR, major pathological response; MSI‐H, microsatellite instability‐high; NMIBC, non‐muscle invasive bladder cancer; NSCLC, non‐small‐cell lung cancer; ORR, objective response rate; OS, overall survival; PCR, pathological complete response; PFS, progression‐free survival; RCC, renal cell carcinoma; RFS, recurrence‐free survival; TNBC, triple‐negative breast cancer; TPS, tumor proportion score; TrAE, treatment‐related adverse events; UC, urothelial carcinoma.

^a^ Both arms received adjuvant pembrolizumab.

**FIGURE 1:**
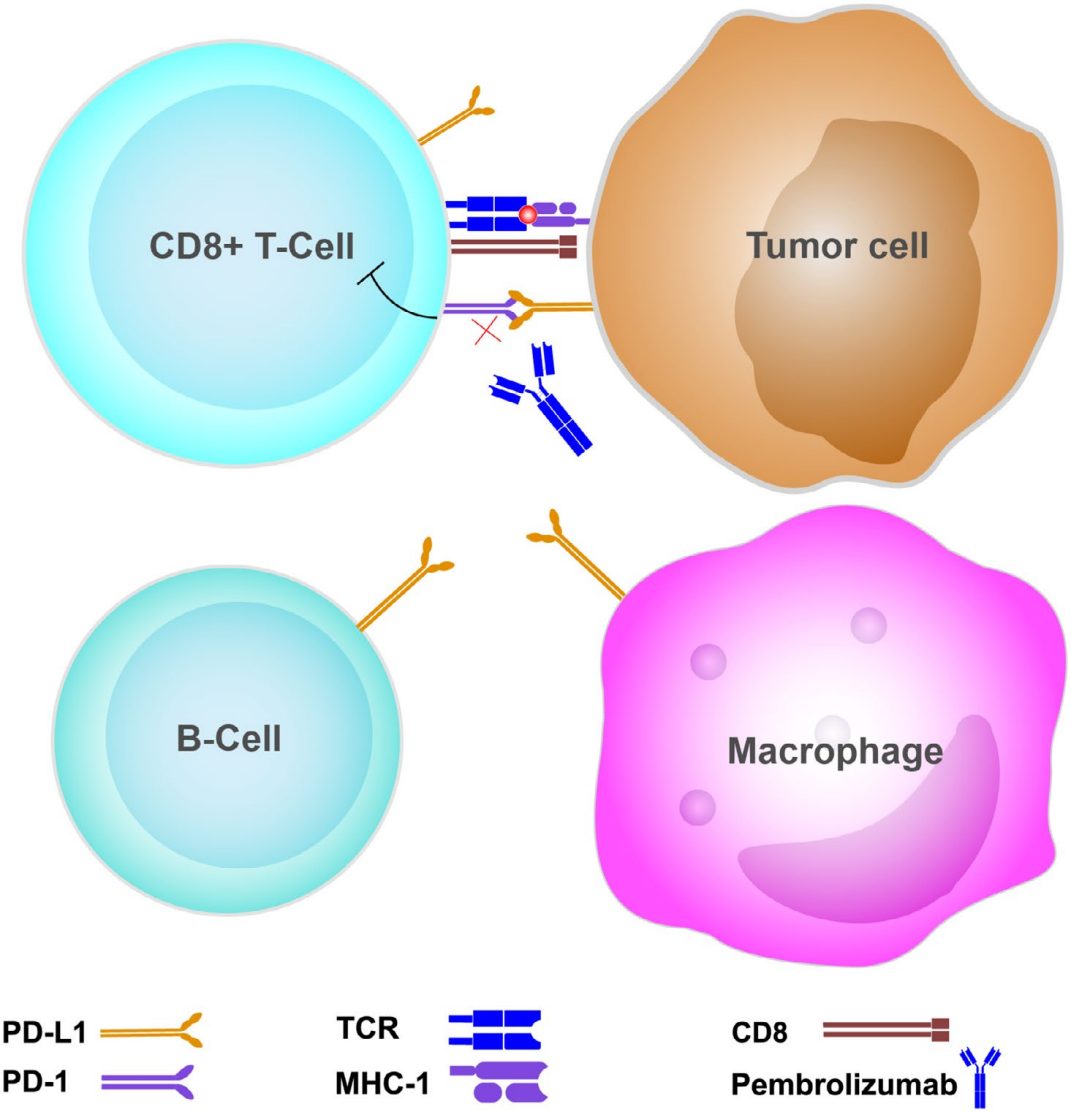
The mechanism of action of anti PD‐1 monoclonal antibodies (i.e., Pembrolizumab). Tumor cells express neoantigens on MHC I. Neoantigen specific CD8+ T cells are recruited and activated by binding its. TCR to neoantigen expressing MHC I. PD‐L1 and PD‐L2 (not shown) are expressed on tumor cells and they inhibit T cells by binding to PD‐1 receptors. Pembrolizumab (a monoclonal antibody against the PD‐1 receptor) impedes PD‐1/PD‐L1/2 interaction and unleashes tumor‐suppressed immune response. PD‐L1 is also normally expressed on lymphocytes and antigen‐presenting cells (APC) like macrophages as depicted PD‐L1/2, programmed death ligand‐1/2; TCR, T‐cell receptor; CD8, cluster of differentiation‐8; PD‐1, programmed cell death receptor‐1; MHC‐1, major histocompatibility complex class‐1.

Immune checkpoints are coinhibitory signaling receptors that normally maintain immune tolerance and control immune responses [55]. PD‐1 is a checkpoint molecule found on the surfaces of activated T‐cells as well as B‐cells and natural killer cells [56]. Its ligands, either PD‐L1 or PD‐L2, are expressed on peripheral normal tissue cells, antigen‐presenting cells (APC), and tumor cells [56]. The PD‐1/PD‐L1_/_2 interaction, however, suppresses T cell‐receptor‐mediated lymphocyte activation and the subsequent cytokine surge [55, 57]. Thus, cancer cells can evade the immune system by expressing PD‐L1 on their surfaces, causing neoantigen‐specific activated T‐cells to anergize and therefore halting the immune surveillance [56]. Anti‐PD‐1 monoclonal antibodies (pembrolizumab, nivolumab, retifanlimab, dostarlimab, and cemiplimab) unleash deactivated immune cells by blocking the inhibitory PD‐1/PD‐L1 interaction [1], and thus reinvigorating immune response against tumor cells (Figure 1).

The tumor microenvironment, which represents the tumor‐immune milieu in which all these interactions occur, is highly heterogenic and dynamic in nature [55]. Therefore, clinical responses to PD‐1 blockade differ among patients, which reflects the need for surrogate biomarkers that predict the efficacy of such therapy in different patient subgroups [58], given the high costs and possible immune‐related adverse events that come along with immunotherapy. Antigenicity biomarkers, including high microsatellite instability (MSI‐H) and high tumor mutation burden (TMB), and inflammatory biomarkers of the tumor microenvironment, including PD‐L1 expression and T‐cell inflamed gene expression profile (GEP) appeared as rational and promising options [58]. Combined positive score (CPS) is determined as the number of cells that express PD‐L1 on their surfaces, including tumor cells as well as surrounding immune cells, divided by the sum of all tumor cells multiplied by 100, while tumor proportion score (TPS) is the ratio of tumor cells that express PD‐L1 [59]. Interestingly, consideration of immune cells PD‐L1 expression can influence the predictive value of PD‐L1 expression as an efficacy biomarker in certain cancers [59], which reflects the complexity of both the tumor microenvironment and the drug mechanism of action (Figure 1). The complex interactions of tumor and immune cells within the tumor microenvironment, along with the great intra‐ and inter‐tumor heterogeneity, limit the development of reliable predictive biomarkers that could be implemented clinically [60]. A combination biomarker approach might help overcome these limitations [60]. Circulating tumor DNA has provided a real‐time and dynamic marker of response to immunotherapy with both prognostic and predictive value [61]. It can indicate response early during treatment when imaging results are still equivocal or misleading [62]. Such biomarker of molecular response can support early therapeutic decisions. Radiological tests also play a significant role in assessing response to immunotherapy using both morphologic and metabolic criteria [63]. Parameters of 2‐deoxy‐2‐[^18^F]fluoro‐D‐glucose positron emission tomography/computed tomography can have prognostic value and are especially important for assessing patients with non‐small‐cell lung cancer and advanced melanoma [63]. Extensive discussion of predictive biomarkers is beyond the scope of this review.

The recommended dose of pembrolizumab is 200 mg every 3 weeks or 400 mg every 6 weeks [64, 65]; however, there has been a debate over the cost‐effectiveness of the fixed dosing approach compared with a weight‐dependent dose of 2 mg/kg every 3 weeks or 4 mg/kg every 6 weeks [66]. Indeed, the weight‐dependent dosing approach can lead to considerable savings in treatment costs and provides similar exposures to pembrolizumab compared with the fixed dose [67, 68]. On the other hand, a fixed dosing regimen appears to be practically more convenient and is the currently approved practice in the US.

The unique immune‐dependent mechanism of action of anti‐PD‐1 antibodies resulted in a proportionately unique set of adverse events collectively termed as immune‐related adverse events (irAE), which are somewhat similar to autoimmune diseases. Commonly observed irAE include: hyperthyroidism, hypothyroidism, hypophysitis, pneumonitis, colitis, and hepatitis among others [69]. The management of irAE is determined by their severity; however, the majority of these symptoms are reversible, and some do not require interventional treatment. Immunosuppression with corticosteroids can be considered as it did not interfere with immunotherapy in retrospective studies [70].

In recent years, an expanding body of evidence has rapidly evolved at a fast pace to study the benefit of these new drugs in various cancers and different combinations. In this review, we outline the role of pembrolizumab in the management of eight highly‐prevalent cancers and discuss how it has revolutionized cancer therapy in terms of durability and survival rates in cancers that were previously associated with short life expectancy and poor quality of life.

## 2 Triple‐Negative Breast Cancer

Triple‐negative breast cancer (TNBC) is marked by the paucity of expression of human epidermal growth factor receptor 2 (HER‐2), as well as estrogen and progesterone receptors on tumor cells [71]. However, elevated expression of PD‐L1 on tumor cell surfaces [72] and infiltration of tumor‐infiltrating lymphocytes (TIL) [73] suggested possible therapeutic effects for immune checkpoint inhibitors (ICI) and led to further investigations. Randomized clinical trials have demonstrated the efficacy of pembrolizumab in both early and metastatic TNBC. In the KEYNOTE‐522 randomized controlled trial [6], among patients with early TNBC, investigators reported a significant increase in the proportion of pathological complete response (pCR) in patients who received pembrolizumab with platinum‐containing chemotherapy prior to surgery, without an increase in chemotherapy‐related toxic effects. Nevertheless, efficacy correlations with tumor PD‐L1 expression could not be established [6]. These findings led to the FDA approval of neoadjuvant pembrolizumab combined with chemotherapy and then given as monotherapy after surgery for this group of patients [74]. The rationale for the immuno‐chemotherapy combination arises from the synergistic effect of both therapies [57]. As immune checkpoint inhibition may elicit a robust anti‐cancer immune response to tumor‐specific antigens released after chemotherapy. Interestingly, a clinically applicable response classifier (ImPrint) was developed using transcriptome microarray data from pre‐treatment biopsies of breast cancer patients enrolled in the I‐SPY2 trial [75]. A refined version of ImPrint was recently developed for TNBC (ImPrintTN), predicting pCR rates of 71% in ImPrintTN^+^ versus 21% in ImPrintTN^−^, and is currently being validated in the I‐SPY2.2 trial [76].

In addition to early‐stage related benefits, pembrolizumab showed considerable anti‐tumor activity with manageable toxic effects in metastatic TNBC [77, 78, 79]. For example, in patients with advanced TNBC and a PD‐L1 expression of CPS ≥ 10, the pembrolizumab‐chemotherapy combination resulted in longer progression‐free survival (PFS) and overall survival (OS) than with chemotherapy alone [median OS: 23.0 months versus 16.1 months; hazard ratio for death (95% confidence interval): 0.71 (0.54–0.93)]. However, no significant OS benefit was found in the intention‐to‐treat population [8, 80]. This elucidates the importance of PD‐L1 expression in the prediction of anti‐PD‐1 immunotherapy efficacy in metastatic TNBC patients [81].

The role of immunotherapy in the management of breast cancer beyond the triple‐negative subtype is still limited and the research is ongoing [82]. Most recently, pembrolizumab has shown promising results in early‐stage, high‐risk, estrogen receptor‐positive/human epidermal growth factor receptor 2‐negative (ER^+^/HER2^−^) breast cancer [83]. The pCR rate was significantly higher in the pembrolizumab‐chemotherapy neoadjuvant group (24.3%; 95% confidence interval (CI): 21.0%–27.8%) compared with the placebo‐chemotherapy group (15.6%; 95% CI: 12.8%–18.6%). Interestingly, pCR benefit was more evident in the PD‐L1 CPS ≥ 1 subgroup and the low ER positivity (< 10%) subgroup. This response pattern could be attributed to the biological similarities of low ER^+^/HER2^−^ breast cancer and TNBC. However, confirmation of higher cPR rates in low ER patients is required due to small subgroup size (77 patients in both arms combined).

### 2.1 Non‐Small‐Cell Lung Cancer

Lung cancer is responsible for the highest cancer‐related mortality, making up to 19% of cancer deaths worldwide [84], and causing more deaths in the United States than colorectal, breast, and prostate cancers combined [85]. It is one of the most commonly diagnosed cancers in the United States, coming second to prostate and breast cancer in men and women, respectively [85]. Non‐small‐cell lung cancer (NSCLC) is the most prevalent class of lung cancer, representing nearly 85% of all diagnosed lung cancer cases [86].

Results from the phase III KEYNOTE‐024 led to pembrolizumab approval as first‐line therapy for patients with previously untreated advanced NSCLC with a TPS ≥ 50% but without *EGFR*/*ALK* aberration as it significantly improved PFS and OS compared with standard platinum‐based chemotherapy [10, 87]. Of interest, the addition of ipilimumab (an anti‐CTLA4 monoclonal antibody) to pembrolizumab in this group of patients did not improve efficacy outcomes and even resulted in worse adverse events [88]. Furthermore, The indication of first‐line pembrolizumab monotherapy can be extended to patients with low PD‐L1 TPS of ≥ 1%; however, its effect was greatest in patients with TPS ≥ 50% [12].

Combinations of pembrolizumab with chemotherapy have also been studied in prospective clinical trials. One phase III clinical trial found that adding pembrolizumab to standard chemotherapy consisting of pemetrexed and platinum‐based drugs can prolong OS and PFS in patients with metastatic nonsquamous NSCLC who did not receive prior therapy and were negative for *EGFR* and *ALK* mutations [14]. Interestingly, the survival benefit of this combination was reported in all subgroups of PD‐L1 TPS, including patients with scores less than 1%. Moreover, the addition of pembrolizumab did not increase chemotherapy‐associated adverse events, and the incidence of irAEs related to pembrolizumab was not higher than that of pembrolizumab alone [14]. Comparable results were found in squamous NSCLC patients [16]. There is a lack of evidence that compares the benefit of the pembrolizumab‐chemotherapy combination with that of pembrolizumab monotherapy in patients with metastatic NSCLC; however, an indirect comparison via networking meta‐analysis reported significant PFS benefit and similar OS with the addition of chemotherapy [89]. This suggests that combination therapy can be considered if the prevention of rapid progression is indicated [89]. Generally, the pembrolizumab‐chemotherapy combination has a higher efficacy than that of chemotherapy alone or other ICI combinations or monotherapies [90]. In addition to the efficacy of pembrolizumab in first‐line settings, it has also provided a durable therapeutic option for previously treated patients who have progressed after platinum‐chemotherapy or a tyrosine kinase inhibitor in *EGFR*/*ALK* mutated patients and are PD‐L1 positive, leading to a significantly prolonged OS in these groups of patients [18]. However, a recent phase III trial found that pembrolizumab should not be used in combination with pemetrexed and platinum chemotherapy in *EGFR*‐mutant metastatic NSCLC who have progressed on first‐line tyrosine kinase inhibitor therapy, as it did not provide OS and PFS benefits compared with chemotherapy alone [91].

In patients with stage IB‐IIIA resectable NSCLC who may or may not have received standard adjuvant chemotherapy after complete surgical resection, pembrolizumab resulted in a significantly longer disease‐free survival compared with placebo irrespective of PD‐L1 expression [20], which supported the integration of pembrolizumab as an adjuvant monotherapy in the management of resectable NSCLC. No significant benefit of pembrolizumab in the PD‐L1 TPS ≥ 50% population was observed, which remains to be evaluated at longer follow‐up analysis. Moreover, a perioperative approach of neoadjuvant pembrolizumab combined with platin‐based chemotherapy followed by surgery and adjuvant immunotherapy has significantly improved event‐free survival (events being: progression, recurrence, or death) in patients with resectable stage II or III NSCLC compared with neoadjuvant chemotherapy alone followed by surgery [22]. This elucidates the importance of neoadjuvant pembrolizumab as a potential therapy for the management of patients with resectable NSCLC. There is sparse evidence investigating PD‐1 blockade as first‐line therapy for *EGFR* mutant NSCLC patients, with only two prospective studies conducted to date to investigate pembrolizumab [92, 93]. The multiplicity of potential biomarkers that have been established (PD‐L1 expression, TMB, and dMMR status), as well as the effective integration of pembrolizumab in the first‐line treatment of *BRAF* mutant melanoma patients, should prompt further investigations in *EGFR* mutant treatment‐na*ï*ve NSCLC patients.

### 2.2 Melanoma

Melanoma, an aggressive skin cancer with metastatic tendency, is increasing in incidence worldwide, which represents a global healthcare burden [94]. Early detection and prompt resection of the tumor are the definitive factors for a favorable prognosis. Immunotherapy has revolutionized the systemic therapy of melanoma, whether given as adjuvant, neoadjuvant therapy, or to patients with unresectable or metastatic melanoma.

In the adjuvant setting, pembrolizumab resulted in a notably longer recurrence‐free survival (RFS) and distant metastasis‐free survival, and a predictable safety profile compared with placebo in patients with resected stage III melanoma regardless of PD‐L1 tumor expression [24, 25]. Similar results were found in resected stage IIB and IIC melanomas [27]. However, the OS benefits of adjuvant pembrolizumab remain uncertain. In comparison with the former standard adjuvant immunotherapies (IFNα‐2b or ipilimumab), pembrolizumab significantly improved RFS but not OS and resulted in fewer treatment‐related adverse events, which supports its application as adjuvant therapy in melanoma patients who have a high risk for recurrence [95]. Of note, RFS was longer in PD‐L1 positive melanomas suggesting potential predictive value that needs further investigation [95].

The effect of anti‐PD‐1 antibodies depends on the antitumor activity mediated by tumor‐infiltrating T‐cells; thus, surgical resection of the tumor with the lymphocyte infiltrate contained in it would limit the utility of PD‐1 blockade [96]. This elucidates the rationale for the neoadjuvant administration of anti‐PD‐1 therapy. In a recent phase II trial, patients with high‐risk, resectable stage III or IV melanoma who received both neoadjuvant and adjuvant pembrolizumab therapy had longer event‐free survival compared to those who received adjuvant therapy alone without increasing the incidence of surgery‐related adverse events [96].

In the metastatic setting, ipilimumab was initially considered the standard‐of‐care treatment for patients with advanced unresectable melanoma. However, pembrolizumab significantly prolonged PFS and OS in this group of patients with fewer high‐grade irAE as compared to ipilimumab [29]. Interestingly, OS benefit was observed in both *BRAF* mutant and wildtype subgroups. Furthermore, in patients with advanced unresectable melanoma who had either progressed on ipilimumab or a BRAF or MEK inhibitor (if *BRAF* ^*V600E/K*^ mutant), pembrolizumab reduced the risk of disease progression as compared with the standard‐of‐care chemotherapy [97]. As such, the current standard of care involves the use of pembrolizumab over the use of ipilimumab monotherapy. However, the combination of ipilimumab and nivolumab remains the standard first‐line regimen for newly diagnosed patients with metastatic melanoma who are fit and eligible for this combination, and ICI monotherapy with pembrolizumab remains an option, especially for those who are at a higher risk of ICI combination intolerance [98].

In patients with *BRAF* ^*V600E/K*^ mutant advanced melanoma, the addition of pembrolizumab to BRAF and MEK inhibitors substantially improved PFS and OS and resulted in more durable responses [99]. The enhanced effect of this combination is explained by the fact that BRAF and MEK inhibitors are associated with an increased expression of melanoma antigens and a denser intratumoral CD8+ T‐cell infiltrate [100]. Moreover, high expression levels of the immunosuppressive ligand PD‐L1 suggest the synergistic effect of anti‐PD‐1 antibodies with the exhausted lymphocytic infiltrate elicited by BRAF inhibition [100]. Combination therapy of standard‐dose pembrolizumab and reduced‐dose ipilimumab has emerged, trying to improve the efficacy of anti‐PD‐1 monotherapy with a modest increase in toxicity for advanced melanoma patients [101], and resulted in a tolerable toxicity profile and considerable anti‐tumor activity. Similarly, this combination demonstrated significant antitumor activity and was well tolerated in advanced melanoma patients who were refractory to previous therapy with anti‐PD‐1 and PD‐L1 antibodies [102]. Trials directly comparing anti‐PD‐1/anti‐CTLA‐4 antibody combinations with ipilimumab monotherapy in anti‐PD‐1/L1 antibody–refractory patients are still needed to highlight the role of these combinations in melanoma. The integration of neoadjuvant pembrolizumab in the management of resectable melanoma awaits phase III confirmatory trials and systematic appraisal of current evidence.

### 2.3 Renal Cell Carcinoma

With an approximate mortality rate of 3.5 per 100 000, renal pelvis and kidney cancer represents 4% of all new cancer cases in the USA [85]. Renal cell carcinoma (RCC) is the most prevalent among all urogenital cancers and is more often observed in males than females. Other risk factors include hypertension, obesity, smoking, and chronic kidney disease [103]. Pembrolizumab, either as monotherapy or in combination with targeted treatments like axitinib or lenvatinib, has changed the landscape of RCC treatment.

Based on molecular and histological subtypes, clear cell renal cell carcinoma (ccRCC) is the most prevalent subtype of RCC [103]. The phase III CLEAR study concluded that first‐line pembrolizumab plus lenvatinib (a multikinase inhibitor) significantly improved OS versus sunitinib for advanced ccRCC patients irrespective of sarcomatoid features, metastatic lesions at baseline, or prior nephrectomy, resulting in the FDA approval of this treatment [32, 104]. It is worth mentioning that the ipilimumab plus nivolumab combination has shown outstanding results in patients with poor or intermediate‐risk metastatic ccRCC [105]. Another phase III trial, KEYNOTE‐426, has demonstrated that first‐line axitinib plus pembrolizumab resulted in longer OS and PFS in comparison with sunitinib for metastatic ccRCC thus supporting the use of axitinib with pembrolizumab as the standard therapy for previously untreated advanced ccRCC patients and leading to the FDA approval of this combination [34, 106].

The standard first‐line therapy for ccRCC patients consists of immunotherapy‐based combinations such as pembrolizumab with lenvatinib. Nevertheless, these combinations are not well studied in advanced non‐clear cell RCC (nccRCC) for which cabozantinib is the preferred regimen [107]. In this regard, a phase II single‐arm trial KEYNOTE‐B61 aims to illustrate the potential benefit of such combination in treatment‐na*ï*ve advanced/metastatic nccRCC patients. So far, findings from this study support the first‐line administration of the pembrolizumab plus lenvatinib combination in this group of patients [108]. The single‐arm phase II trial, KEYNOTE‐42, investigated the efficacy of first‐line pembrolizumab in patients with previously untreated advanced ncc‐RCC and has shown encouraging anti‐tumor activity in the overall nccRCC cohort, with a safety profile similar to that observed in other tumor types [109]. Better responses were observed in papillary nccRCC compared to chromophobe nccRCC, leading pembrolizumab to be introduced as a therapeutic option for patients with papillary nccRCC [109, 110]. Furthermore, a phase I/II study NCT03149822 yielded encouraging efficacy and safety results for cabozantinib plus pembrolizumab combination in clear cell and non‐clear cell mRCC patients, which is comparable to other available checkpoint inhibitor/tyrosine kinase inhibitor combinations, warranting further prospective evaluation [111].

Oligometastatic ccRCC is defined as the presence of limited (typically 1 to 5) metastases [112]. The single‐arm phase I/II trial RAPPORT clarified that applying stereotactic ablative body radiotherapy (SABR) followed by short‐course pembrolizumab in patients with oligometastatic ccRCC was well tolerated, with encouraging PFS and excellent local control. However, the selected patient population and a single‐arm design are major limitations of this study. Accordingly, further investigation is needed [113].

The standard of care for non‐metastatic ccRCC is surgery. However, nearly 50% of patients relapse later to surgery. Thereupon, the ongoing phase II randomized trial NAPSTER suggests that neoadjuvant SABR will improve disease outcomes either alone or when applied with pembrolizumab therapy. In this case, the use of one or both arms as a neoadjuvant therapy before surgery in high‐risk patients could be justified in the future [114].

In the adjuvant setting, updated results from the randomized phase III study KEYNOTE‐564 showed a considerable DFS benefit of post‐nephrectomy adjuvant pembrolizumab in ccRCC patients who had an increased risk of recurrence [35]. These findings supported the FDA approval of adjuvant pembrolizumab for the treatment of patients with RCC at high or intermediate‐high risk of recurrence after surgery [36, 114]. However, the efficacy of neoadjuvant pembrolizumab in those patients is to be investigated in future trials. In addition, biomarkers are still needed to predict the efficacy of adjuvant pembrolizumab therapy.

### 2.4 Bladder Cancer

Bladder cancer (BC) accounts for nearly 4% of all malignancies, finding its place among the 10 most common cancers and being a significant cause of malignancy‐related deaths [85]. The therapeutic benefits of ICI such as pembrolizumab depend on the phase during which they are being applied, as well as PD‐L1 expression [115].

For advanced/unresectable or metastatic urothelial carcinoma (UC), the KEYNOTE‐045 phase III study found that pembrolizumab resulted in a higher OS than second‐line chemotherapy in patients refractory to previous platinum‐based therapy, making it the standard of care for this group of patients [37, 38]. Meanwhile, the phase II single‐arm trial KEYNOTE‐052 has shown a durable benefit of first‐line pembrolizumab therapy for cisplatin‐ineligible patients. These results granted pembrolizumab an accelerated approval by the FDA in this setting [37, 38]. Furthermore, the phase III trial EV‐302 showed that applying pembrolizumab with enfortumab vedotin resulted in a significantly longer PFS and OS in treatment‐naïve patients with locally advanced or metastatic UC in comparison with platinum‐based chemotherapy, which led to the recent approval of this combination [41, 42]. This came a while after the phase III trial EV‐103 (cohort K) established the superiority of the abovementioned combination over enfortumab vedotin monotherapy, as it resulted in a higher objective response rate and a longer duration of response in cisplatin‐ineligible patients, giving rise to the accelerated approval of this therapy [39, 40].

Promising antitumor efficacy has been demonstrated by the phase II single‐arm trial KEYNOTE‐057 for patients with non‐muscle invasive bladder cancer (NMIBC) who did not respond to intra‐lesional BCG [43]. To increase safety among those patients, a phase I study NCT02808143 concluded that injecting pembrolizumab directly into the bladder can be a safe and durable alternative. however, further investigation is needed [116]. It is worth mentioning that the FDA has recently approved pembrolizumab monotherapy for patients with BCG‐refractory carcinoma in situ who declined surgery or did not qualify for cystectomy [117].

The clinical benefit of pembrolizumab has also been under investigation in patients with muscle‐invasive bladder cancer (MIBC). In this regard, the phase II study PURE‐01 has proved the efficacy of neoadjuvant pembrolizumab therapy followed by radical cystectomy (RC) for patients with MIBC. Of note, PD‐L1 expression was a strong predictor of post‐RC response in this study [118]. At present, KEYNOTE‐866 is a phase III trial being held to elucidate the role of perioperative pembrolizumab added to conventional neoadjuvant chemotherapy compared with neoadjuvant chemotherapy alone in formerly untreated cisplatin‐eligible patients with MIBC who qualify for RC with dissection of pelvic lymph nodes [119]. As for cisplatin‐ineligible MIBC patients, another phase III trial KEYNOTE‐905/EV‐303 is currently underway to investigate the efficacy of perioperative pembrolizumab whether given alone or with enfortumab vedotin, compared with RC and dissection alone [119]. For patients with non‐metastatic MIBC who desire bladder preservation, a phase III study KEYNOTE‐922 is now ongoing to investigate the safety and efficacy of adding pembrolizumab to chemoradiation [120]. Despite this progress, we still lack sufficient biomarkers to determine optimum treatment options for different patient subgroups.

### 2.5 Head and Neck Cancer

Head and neck squamous cell carcinoma (HNSCC), which arises from mucosal surfaces of the oral cavity, sinonasal cavity, oropharynx, hypopharynx, and larynx, is the eighth most common cancer in men in the United States [85, 121]. It is closely related to the heavy consumption of tobacco and alcohol, as well as oncogenic HPV oropharyngeal infection [121]. The standard first‐line treatment for recurrent or metastatic disease (R/M HNSCC) has been the combination of cetuximab, platinum, and fluorouracil [59]. Following the durable anti‐tumor activity and tolerable safety profile of pembrolizumab in heavily pre‐treated patients who suffer R/M HNSCC, it was approved for those with metastatic or recurrent disease that has progressed on platinum‐containing chemotherapy and for all PD‐L1 expression subgroups [59]. However, the KEYNOTE‐055, a single‐arm, phase II study, was the first study to assess the efficacy and safety of pembrolizumab in R/M HNSCC that have progressed on both platinum and cetuximab [122] and it supports the effectiveness of pembrolizumab therapy in this setting. The phase III KEYNOTE‐040 trial proved the superiority of pembrolizumab over the standard second‐line therapy (either methotrexate, docetaxel, or cetuximab) in patients with R/M HNSCC that had progressed on platinum‐containing treatment as it resulted in a significantly longer OS and a better safety profile [45]. Thus proving the efficacy and safety of pembrolizumab as second‐line therapy in R/M HNSCC. Moreover, pembrolizumab monotherapy was found superior to the conventional first‐line cetuximab‐chemotherapy combination in R/M HNSCC patients who are PD‐L1 positive, and regardless of PD‐L1 expression when combined with platinum‐containing treatment and fluorouracil [123]. Nevertheless, trials comparing pembrolizumab monotherapy with pembrolizumab‐chemotherapy combination in R/M HNSCC patients have not been conducted to date. OncoPrism‐HNSCC is an RNA‐based clinical assay that has been recently validated to predict disease control in response to anti‐PD‐1 ICIs in patients with R/M HNSCC [124].

About 60% of all HNSCC diagnosed patients have locally or regionally advanced disease (LA‐HNSCC) [125]. Treatment options for these patients include either definitive chemoradiotherapy (CRT) or surgery followed by radiation with consideration of chemotherapy. The efficacy of pembrolizumab in R/M HNSCC patients and its tolerable safety profile warranted further investigations in LA‐HNSCC patients. One phase Ib, single‐armed trial concluded that pembrolizumab combined with cisplatin‐based CRT is safe in patients with LA‐HNSCC [126]. However, a larger, randomized, phase III trial is intended to elucidate the exact role of such combination in LA‐HNSCC [125].

Pembrolizumab could also be integrated into the surgical management of LA‐HNSCC. For instance, the dual neoadjuvant therapy of pembrolizumab added to chemotherapy improved laryngeal function preservation rate [127]. Uppaluri et al. found that neoadjuvant pembrolizumab before surgery was safe and did not interfere with surgery or adjuvant chemotherapy and provided the first evidence that pathologic response observed in the surgical specimen in response to neoadjuvant pembrolizumab may be a predictive biomarker of survival benefit [128]. In line with this, Wise‐Draper et al. further confirmed the notion that pathologic response is associated with improved disease‐free survival [129]. However, results from the two aforementioned studies should only be interpreted with discretion as they had several important limitations. One phase III controlled trial (NCT 03765918) is underway to overcome these limitations and yield applicable conclusions regarding the survival benefit of perioperative pembrolizumab [130]. As outlined above, more rigorous research is awaited to clarify the potential role of pembrolizumab (whether as neoadjuvant/adjuvant therapy or in combination with definitive CRT) in the management of LA‐HNSCC patients.

### 2.6 Endometrial Cancer

Uterine corpus cancer is the fourth most common diagnosed cancer in women [85], and its incidence and mortality rates are continuing to increase. Even though early‐stage endometrial cancer (EC) has a favorable 5‐year survival rate of 96%, the prognosis of metastatic disease remains poor at 20% [131]. Of note, survival for women with uterine corpus cancer has decreased over the past 4 decades, being the only cancer with such a survival pattern [85].

Platinum‐based chemotherapeutic combinations such as carboplatin/paclitaxel or carboplatin/docetaxel, if paclitaxel is contraindicated, are preferred as first‐line therapy for advanced/recurrent EC [132]. However, treatment options for patients who have progressed on these regimens have been limited and have had poor clinical efficacy. In patients with PD‐L1 positive advanced EC who have progressed after standard therapy, the phase Ib KEYNOTE‐028 study proved pembrolizumab to have a tolerable safety profile and durable antitumor effect as it resulted in a partial response in three patients and prevented disease progression in three other patients [133]. In line with the tumor agnostic approval of pembrolizumab for MSI‐H/dMMR solid tumors that have progressed after prior therapy and have no satisfactory alternative treatment options [5], pembrolizumab also demonstrated a durable antineoplastic effect with manageable adverse events in advanced EC patients who have failed to improve on prior therapy and have MSI‐H/dMMR tumors [49]. Of interest, higher objective response rates were observed in patients who had previously received fewer lines of therapy, which may indicate an advantage for the early administration of pembrolizumab therapy [49] Moreover, a prognostic significance of the mechanism of microsatellite instability was first investigated by Bellone et al. [134]. They found that patients with recurrent EC who had somatic acquired MMR gene mutations (Lynch‐like patients) had higher tumor mutation burden (TMB) and longer PFS and OS in response to pembrolizumab compared with sporadic patients who had homozygous methylation of the MLH1 gene promoter. This offers an additional predictive criterion to identify the best responders to pembrolizumab. However, only about 16% of recurrent ECs are MSI‐H [135], and pembrolizumab is not as effective for microsatellite stable (MSS) tumors. A promising antitumor activity for lenvatinib combined with pembrolizumab was observed in mismatch‐repair‐proficient (pMMR) advanced EC patients after progression on first‐line chemotherapy and led to its accelerated approval in this group of patients [135]. The phase III Study 309‐KEYNOTE‐775 trial further confirmed the previous findings and reported longer PFS and OS values for this combination compared with chemotherapy in patients with pMMR EC who had progressed on prior platinum‐based therapy [51].

Incorporation of pembrolizumab as a frontline therapy for advanced or recurrent EC patients in combination with the standard chemotherapy has improved clinical outcomes as PFS rates were higher than those of chemotherapy alone irrespective of MMR status [136]. Trials are still needed to investigate the possible efficacy of first‐line pembrolizumab as a monotherapy in patients with advanced or recurrent dMMR EC and compare it with the established benefit of pembrolizumab‐chemotherapy combination [136]. The phase III trial NCT03884101 is currently underway to prove the expected superiority of pembrolizumab plus lenvatinib combination over the standard chemotherapy as first‐line treatment for advanced or recurrent EC patients [137]. This reflects a rising tendency to incorporate pembrolizumab into first‐line regimens of advanced EC, opposed by the lack of trials that investigate its efficacy in earlier EC stages.

### 2.7 Colorectal Cancer

Colorectal cancer (CRC) accounts for almost 8% of all cancer cases in the USA and approximately 8.7% of all cancer‐related deaths, being the third lethal cancer in both sexes [85]. MSI is a major predictive biomarker for pembrolizumab‐based immunotherapy of CRC; other biomarkers include PD‐L1 expression, TIL, and TMB [138]. About 15% of CRCs are MSI‐H, concealing deficient DNA mismatch repair. ICIs, such as pembrolizumab, have altered the treatment of this type of CRC [139]. The phase III randomized trial KEYNOTE‐177 reported higher PFS with first‐line pembrolizumab versus standard chemotherapy in metastatic dMMR/MSI‐H CRC, with a durable efficacy and fewer adverse events [53]. These results led to the FDA approval of pembrolizumab as a frontline treatment for unresectable or metastatic MSI‐H/dMMR CRC [54, 140].

In the second‐line setting, a phase II trial KEYNOTE‐164 showed feasible antitumor efficacy of pembrolizumab in treatment‐refractory patients with MSI‐H/dMMR unresectable advanced or metastatic CRC who had received at least two former lines of standard treatment, resulting in a longer OS with a reasonable safety profile [3, 141]. An ongoing phase II trial KEYSTEP‐008 aims to test the efficacy and safety of pembrolizumab‐based combinations compared with pembrolizumab monotherapy in both treatment‐naïve and previously treated stage IV MSI‐H/dMMR CRC patients [142].

Pembrolizumab is not well studied as a neoadjuvant therapy for CRC. In this regard, a phase II single‐center trial (NCT04082572) studied neoadjuvant pembrolizumab in high‐risk resectable/localized non‐resectable MSI‐H/dMMR CRC and concluded that preoperative pembrolizumab had limited adverse effects with a robust clinical activity, as well as a high pCR rate in patients who underwent surgical resection. However, this study is single‐institutional and has a small size, so larger prospective trials are needed [143].

As mentioned, pembrolizumab has been proven to be of benefit in MSI‐H/dMMR CRC; however, most CRC patients have MSS/pMMR CRC, which usually does not respond to PD‐1 inhibitors like pembrolizumab. Even though efforts have been made trying to boost the anti‐tumor features of PD‐1 blockage in this group of patients, no significant improvement has been observed to date [144, 145].

It is worth mentioning that pembrolizumab has also been recently studied in resectable gastric and gastroesophageal junction (GEJ) adenocarcinoma in combination with first‐line neoadjuvant and adjuvant chemotherapy [146]. Although pembrolizumab did not add event‐free survival benefit compared with chemotherapy alone, it improved the pCR. Before that, the FDA has approved pembrolizumab as part of the first‐line combination therapy for HER‐2 negative and HER‐2 positive gastric or GEJ adenocarcinoma [147, 148].

## 3 Conclusions and Future Directions

Although pembrolizumab has changed the treatment paradigms of many prevalent cancers, a gap in the perception of its full role in cancer therapy is still present. Despite the translation of PD‐L1 expression, TMB, and mismatch‐repair status into clinical practice, response inconsistencies remain a challenge for optimum treatment. A thorough research of possible molecular determinants of response is required to better understand the immune evasion mechanisms that may overcome immune checkpoint blockade in certain subsets of patients and define those who are more likely to benefit from such therapy. In the era of precision medicine and next generation sequencing, a genetic profile suggestive of durable response with ICI immunotherapy could represent the next leap in this field. The established benefit of pembrolizumab in metastatic disease warrants a more extensive investigation of its efficacy as a neoadjuvant or adjuvant therapy in surgically managed early‐stage disease. Pembrolizumab has so far optimized survival outcomes for the surgical management of early TNBC and NSCLC in combination with the standard neoadjuvant chemotherapy followed by adjuvant monotherapy. Adjuvant pembrolizumab monotherapy has also yielded favorable results in resectable RCC and melanoma; however, the estimation of OS benefit related to pembrolizumab in the neoadjuvant or adjuvant setting requires longer follow‐up periods to be significantly assessed with future analysis. Synergistic patterns of different ICI combinations should be investigated as novel immunotherapies are emerging. This approach maximizes anti‐tumor benefit and minimizes the tumor's ability to re‐escape immune surveillance and recur; however, it is associated with more serious irAE. Thus, a benefit‐to‐complication balance should be met in future research. In summary, not only has pembrolizumab been a breakthrough in the field of cancer therapy, but it has also started a new era of immunotherapy that its therapeutic horizons are yet to be fully defined.

## Author Contributions

**Raghed Mansour:** conceptualization‐lead, writing – original draft‐lead, writing – review and editing‐lead. **Alghaidaq Shreba:** writing – original draft – equal, writing – review and editing – equal. **Karam Khaddour:** supervision‐supporting, writing – review and editing – lead. **Michael Georgeos:** supervision‐supporting, writing – review and editing‐supporting. **Zuheir Alshehabi:** supervision‐lead, writing – review and editing – supporting.

## Ethics Statement

The authors have nothing to report.

## Conflicts of Interest

The authors declare no conflicts of interest.

## Data Availability

Data sharing are not applicable to this article as no new data were created or analyzed in this study.
